# A Practical Treatise on Fractures and Dislocations

**Published:** 1860-04

**Authors:** 


A Practical Treatise on Fractures and Dislocations. By Frank Hastings Hamilton, M D., Prof of Surgery in the University of Buffalo, etc., etc. Illustrated with 289 Wood Cuts. Philadelphia: Blanchard & Lea. 1860.

This is a large sized octavo volume of 757 pages; and is the most complete special work on Fractures and Dislocations accessible to the American profession. A more extended notice of its contents will be given in our next number.

